# Myosin Phosphatase Is Implicated in the Control of THP-1 Monocyte to Macrophage Differentiation

**DOI:** 10.3390/ijms22052516

**Published:** 2021-03-03

**Authors:** Emese Tóth, Ferenc Erdődi, Andrea Kiss

**Affiliations:** 1 Department of Medical Chemistry, Faculty of Medicine, University of Debrecen, H-4032 Debrecen, Hungary; toth.emese@med.unideb.hu; 2 MTA-DE Cell Biology and Signalling Research Group, University of Debrecen, H-4032 Debrecen, Hungary

**Keywords:** macrophage differentiation, myosin phosphatase, MYPT1, PMA, EGCG, ROCK inhibitor

## Abstract

Monocyte to macrophage differentiation is characterized by the activation of various signal transduction pathways, which may be modulated by protein phosphorylation; however, the impact of protein kinases and phosphatases is not well understood yet. It has been demonstrated that actomyosin rearrangement during macrophage differentiation is dependent on Rho-associated protein kinase (ROCK). Myosin phosphatase (MP) target subunit-1 (MYPT1) is one of the major cellular substrates of ROCK, and MP is often a counter enzyme of ROCK; therefore, MP may also control macrophage differentiation. Changes in MP activity and the effects of MP activation were studied on PMA or l,25(OH)_2_D_3_-induced differentiation of monocytic THP-1 cells. During macrophage differentiation, phosphorylation of MYPT1 at Thr696 and Thr853 increased significantly, resulting in inhibition of MP. The ROCK inhibitor H1152 and the MP activator epigallocatechin-3-gallate (EGCG) attenuated MYPT1 phosphorylation and concomitantly decreased the extent of phosphorylation of 20 kDa myosin light chain. H1152 and EGCG pretreatment also suppressed the expression of CD11b and weakened the PMA-induced adherence of the cells. Our results indicate that MP activation/inhibition contributes to the efficacy of monocyte to macrophage differentiation, and this enzyme may be a target for pharmacological interventions in the control of disease states that are affected by excessive macrophage differentiation.

## 1. Introduction

Monocytes and macrophages are important mediators of innate immune responses and inflammatory processes. Circulating monocytes can differentiate into macrophages when they migrate into tissues or to the sites of inflammation [1]. Differentiation of monocytes to macrophages can be either beneficial or detrimental in disease states including atherosclerosis, liver fibrosis, neurodegenerative diseases, and cancer [2,3]. Therefore, understanding the differentiation process at molecular level and the description of regulatory mechanisms behind may aid in the identification of new therapeutic targets. Protein phosphorylation is one fundamental regulatory mechanism for many cellular processes and signal transduction pathways. The level of protein phosphorylation is determined by the opposing action of protein kinases and phosphatases. Members of these two enzyme families have been implicated in the control of a plethora of cell functions; however, their role in the regulation of signaling pathways during monocyte to macrophage differentiation has not been revealed in every aspect.

THP-1, a human monocytic cell line, is one of the most widely used in vitro models for monocyte to macrophage differentiation. Phorbol-12-myristate-13-acetate (PMA), an activator of protein kinase C (PKC), induces adherence, growth arrest, and differentiation of THP-1 cells into a macrophage phenotype [4]. These PMA-differentiated cells have altered morphology and express macrophage surface markers, including CD14 and CD11b. Moreover, they exhibit altered expression of several cell cycle-related genes, such as upregulation of the cyclin-dependent kinase inhibitor 1 (p21) and decreased level of proliferating cell nuclear antigen (PCNA) [5,6]. 1,25-Dihydroxyvitamin D_3_ (VD_3_) is another agent used to differentiate THP-1 cells into macrophage-like cells; however, VD_3_ treatment leads to a smaller degree of differentiation, with lack of adherence and moderate proliferation inhibition [7,8]. Different extents of differentiation suggest that PMA and VD_3_ act in a different manner; however, the intracellular signaling molecules activated are identical in part. Upregulation of PKC isoenzymes is a common mechanism during both differentiation processes, but distinct isoforms of PKC are implicated [7,9]. Both PMA and VD_3_ trigger the activation of the PI3K/Akt pathway as well as the modulation of MAPK signaling network [9,10,11]. Bhattacharya et al. have demonstrated recently that MAPK-driven signaling pathways direct macrophage differentiation by regulation of cellular actomyosin dynamics [12]. In addition, the activation of Rho-associated kinase (ROCK) also appears to be essential for the completion of differentiation by modulating the phosphorylation, and thereby activation, of LIM kinase, n-cofilin, and 20 kDa myosin light chain (MLC20) [12,13].

Despite the essential contribution of protein kinases, little is known about the functions of protein phosphatases in the control of monocyte to macrophage differentiation. Myosin phosphatase (MP), a holoenzyme complex of protein phosphatase type 1δ (PP1cδ) and the regulatory subunit termed myosin phosphatase target subunit-1 (MYPT1), is one of the counter enzymes of ROCK in the control of the phosphorylation level of several cellular substrates [14,15]. ROCK and MP regulate the phosphorylation state of MLC20 in a cooperative manner. Myosin light chain kinase and ROCK catalyze the phosphorylation of MLC20, while MP is thought to be the major phosphatase involved in the dephosphorylation process [16]. The activity of MP is inhibited by phosphorylation of MYPT1 at Thr696 and Thr853 residues by several kinases including ROCK, which promotes MLC20 phosphorylation that ultimately leads to actomyosin contractility [17]. Inhibition of MP is also associated with cell adhesion, migration, and spreading by regulating cytoskeletal dynamics [18,19,20,21]. Given the role of MP in cell functions related to actomyosin rearrangement, we addressed the question whether MP would act as a regulator during monocyte to macrophage differentiation. Therefore, our aims were to investigate changes in MP activity and the effects of MP activation during chemical stimuli induced differentiation of THP-1 cells to macrophages.

## 2. Results

### 2.1. Differentiation of THP-1 Cells Induced by PMA Is Accompanied by Phosphatase Inhibition

**Figure 1:**
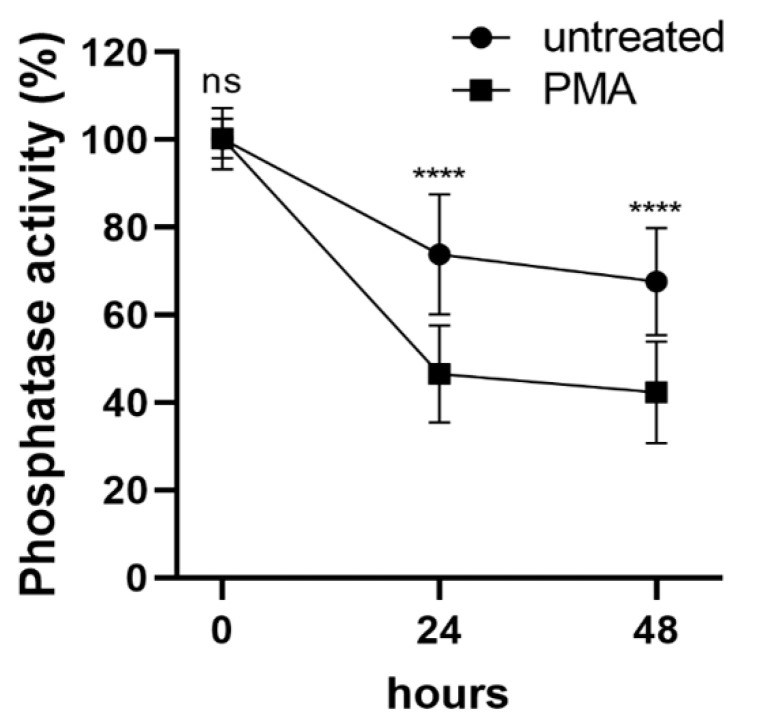
PP1- and PP2A-type phosphatases are inhibited during PMA-induced monocyte differentiation. THP-1 cells were treated with none or 100 nM PMA, then cell lysates were prepared at the indicated times, and phosphatase activity was assayed as described in Materials and Methods. Phosphatase activity of untreated cells at the start of incubations was taken as 100%. Statistical analysis was performed by multiple t-test and Sidak’s multiple comparisons (*n* = 4, **** *p* < 0.0001, ns: not significant).

Protein phosphatase-1 (PP1) and -2A (PP2A) enzymes play important roles in cellular signaling pathways via dephosphorylation of a large number of phosphoproteins [22,23,24]; however, little is known about their function during monocytic differentiation. To determine the change in total PP1 and PP2A activity during PMA-induced differentiation we performed in vitro phosphatase activity assays of cell lysates using phosphorylated 20 kDa myosin light chain as substrate (Figure 1). Phosphatase activity (normalized to protein content) decreased with time in unstimulated THP-1 cells reaching approximately 68% of the initial value after 48 h. However, when THP-1 cells were stimulated with PMA, the decrease in phosphatase activity was even more pronounced, resulting in 46 to 42% residual activity after 24 or 48 h of differentiation, respectively. These data suggest that PMA-induced differentiation of THP-1 cells might be accompanied by inhibition of PP1 and/or PP2A.

### 2.2. Myosin Phosphatase Is Inhibited during PMA- and VD_3_-Induced Differentiation of THP-1 Cells

**Figure 2:**
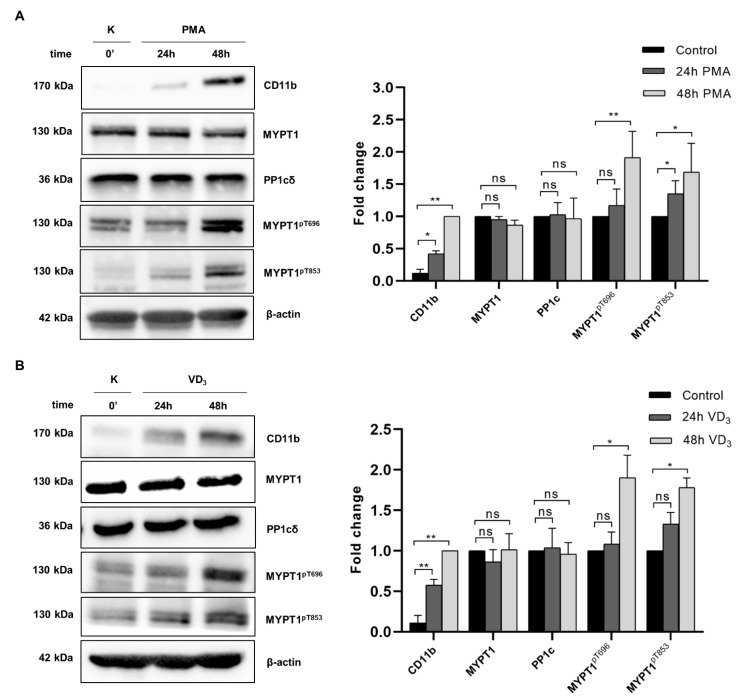
Changes in the expression and phosphorylation of myosin phosphatase subunits during PMA- and VD_3_-induced differentiation. THP-1 cells were stimulated with PMA (**A**) or VD_3_ (**B**) to induce differentiation, then cell lysates were prepared at the indicated times, and expression of CD11b, MYPT1, PP1cδ, MYPT^pT696^, MYPT^pT853^, and β-actin were analyzed by Western blotting. Densitometry analysis was performed with ImageJ to quantify band intensities. CD11b, MYPT1 and PP1cδ were normalized to β-actin, whereas MYPT^pT696^, and MYPT^pT853^ were normalized to total MYPT1. Histograms demonstrate fold changes relative to cells prior to differentiation (for MYPT1, MYPT^pT696^, MYPT^pT853^, and PP1cδ) or after 48 h PMA-treatment (for CD11b). Data were analyzed by two-way ANOVA followed by Dunnett’s multiple comparisons (*n* = 5, * *p* < 0.05, ** *p* < 0.01, ns: not significant).

**Figure 3:**
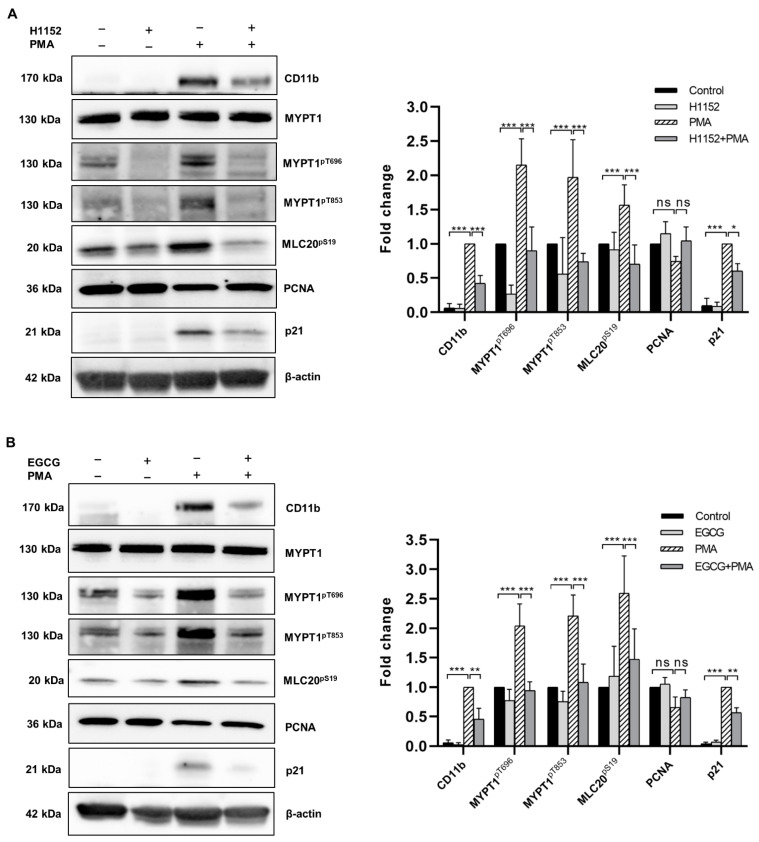
H1152 and EGCG activate myosin phosphatase and attenuate PMA-induced macrophage differentiation. THP-1 cells were pretreated with 10 µM H1152 for 30 min (**A**) or 20 µM EGCG for 1 h (**B**), then treated with PMA for 48 h. Expressions of CD11b, MYPT1^pT696^, MYPT1^pT853^, MLC20^pS19^, PCNA, and p21 were analyzed by Western blotting. The total MYPT1 and β-actin were used as loading controls. Histograms demonstrate fold changes of protein or phosphorylation levels relative to unstimulated cells (for MYPT^pT696^, MYPT^pT853^, MLC20^pS19^, and PCNA) or PMA-stimulated cells (for CD11b and p21). Data were analyzed by two-way ANOVA, followed by Tukey’s multiple comparisons (*n* = 5, * *p* < 0.05, ** *p* < 0.01, *** *p* < 0.001, ns: not significant).

It was shown previously that the RhoA/Rho kinase (ROCK) signaling pathway mediates macrophage differentiation induced by PMA [13]; however, the potential effect of myosin phosphatase (MP), a counter enzyme for ROCK phosphorylation, has not been explored. Therefore, we investigated the activity of MP during the course of macrophage differentiation by analyzing the expression and/or phosphorylation of its subunits using Western blotting (Figure 2A). The expression of the macrophage differentiation-associated surface marker CD11b was detected to test the effectiveness of differentiation, which showed approximately 2- to 4-fold induction upon PMA-stimuli at 24 and 48 h, respectively. Expression of the PP1cδ catalytic and MYPT1 regulatory subunits of MP were not affected by PMA treatment, while a significant increase was observed in the phosphorylation of MYPT1 at both Thr696 (MYPT1^pThr696^) and Thr853 (MYPT1^pThr853^). Enhanced phosphorylation at these residues causes the inhibition of MP, suggesting that PMA-induced macrophage differentiation was accompanied by reduced MP activity. In accordance with MP inhibition, we detected enhanced phosphorylation of the MP substrate 20 kDa myosin light chain (MLC20) in the presence of PMA (see Figure 3A, lane 4).

1,25-Dihydroxyvitamin D_3_ (VD_3_) is another stimulus commonly used to induce differentiation in monocytic cell lines, but the resulting phenotype is notably different. In contrast to PMA stimuli, differentiation of THP-1 cells by VD_3_ is not accompanied by cell adhesion or loss of proliferative capacity, but it results in a similar extent of CD11b expression. To investigate whether MP also plays a role in controlling VD_3_-induced differentiation, we examined the changes in MP activity during this process (Figure 2B). Neither MYPT1 nor PP1cδ protein levels changed when the cells were incubated in the presence of VD_3_ for 24 or 48 h, while the expression of CD11b significantly increased, confirming the efficacy of differentiation. In line with this, phosphorylation of Thr696 and Thr853 residues increased gradually, suggesting that VD_3_-induced differentiation was also coupled with MP inhibition. These data suggest that MP inhibition is associated with both PMA-and VD_3_-induced monocyte differentiation.

### 2.3. Both H1152 and EGCG Pretreatment Activates Myosin Phosphatase and Attenuates PMA-Induced Macrophage Differentiation

Next, we addressed the question if pharmacological activation of MP could interfere with the effects of differentiation induction in THP-1 cells. ROCK is the major kinase that phosphorylates both Thr696 and Thr853 residues; therefore, inhibition of ROCK can attenuate MYPT1 phosphorylation and relieve MP from the inhibition [15]. The selective ROCK inhibitor H1152 was applied to THP-1 cells before the induction of differentiation by PMA, and the phosphorylation of MYPT1 was tested after 48 h. H1152 treatment not only decreased the basal level of MYPT1 phosphorylation in non-differentiated cells (although this tendency of changes was not significant), but it also prevented partially the PMA-induced increase in both MYPT1^pThr696^ and MYPT1^pThr853^ levels (Figure 3A). Phosphorylation at Ser19 of the MP substrate 20 kDa myosin light chain (MLC20^pS19^) markedly increased after 48 h of incubation with PMA, but it decreased significantly in case of H1152-pretreatment correlating with MP activity. In addition, H1152 pretreatment significantly attenuated the expression of CD11b, suggesting that the efficacy of macrophage differentiation was related to the activity of MP. We also analyzed the expression of cyclin-dependent kinase inhibitor 1 (p21) and proliferating cell nuclear antigen (PCNA), two cell cycle regulators known to be responsible for the reduced cell proliferation coupled to the PMA-induced differentiation process [6,25]. In agreement with data from other publications, we observed reduction in the expression of PCNA upon PMA treatment, which was partly prevented when the cells were pretreated with H1152, although these changes in PCNA level were not significant. H1152 also tendentiously suppressed the PMA-stimulated expression of p21.

Alternatively, MP can be activated by enhancing the activity of the phosphatase(s) responsible for the dephosphorylation of inhibitory phosphosites of MYPT1. In this regard, the green tea polyphenol epigallocatechin-3-gallate (EGCG) was shown to induce the dephosphorylation of MYPT1^pT696^ by stimulating PP2A via the 67 kDa laminin receptor (67LR) [26,27]. We have demonstrated recently that the EGCG/67LR/PP2A pathway also functions in THP-1 cells [28]. In the present differentiation model, phosphorylation of both Thr696 and Thr853 was lowered when EGCG was added before stimulating the cells with PMA (Figure 3B). This dephosphorylation-induced activation of MP was followed by reduced phosphorylation of MLC20, lower expression of both CD11b and p21, and a partial restoration of PCNA level.

These data support the hypothesis that activation of MP might counteract with the PMA-induced differentiation process in THP-1 cells.

### 2.4. Activation of Myosin Phosphatase Decreases the Strength of Adherence of Differentiating Macrophages

**Figure 4:**
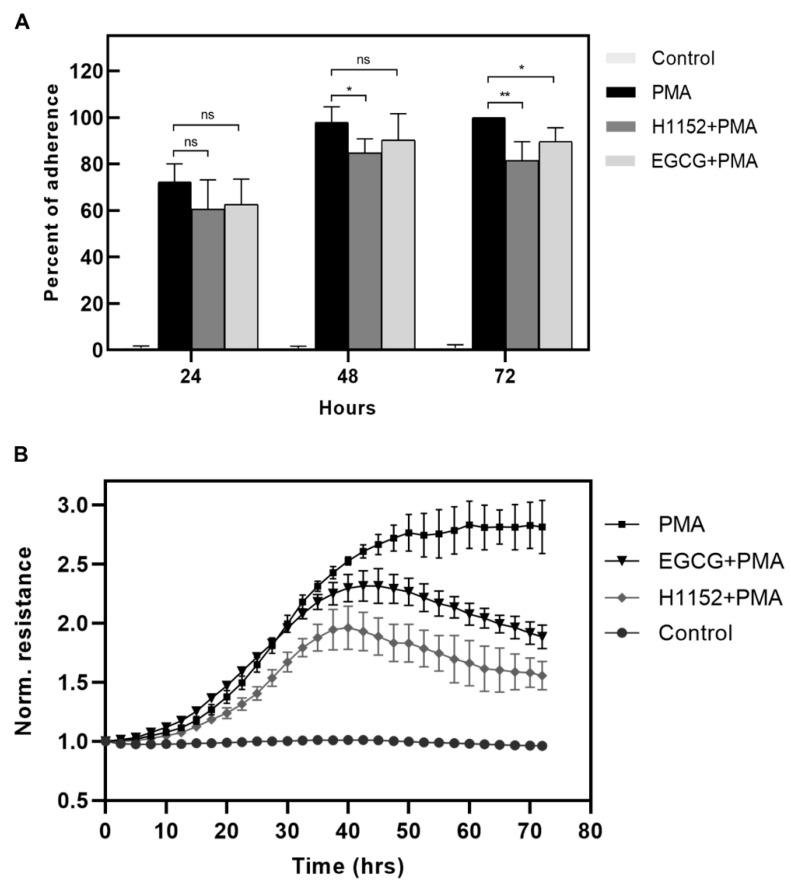
Effect of H1152 and EGCG on the adhesion of THP-1 cells upon PMA-induced differentiation. (**A**) THP-1 cells were pretreated with 10 µM H1152 for 30 min or 20 µM EGCG for 1 h, then stimulated with PMA. Non-adherent cells were removed at the indicated time points, and cells were stained with sulforhodamine B (SRB). The adherence of the cells was determined by quantification of SRB staining and expressed as percentage of PMA-stimulated cells at 72 h. Histograms represent the means of three independent experiments. Multiple t-test. * *p* < 0.05, ** *p* < 0.01. (**B**) THP-1 cells were treated as indicated. Cell adherence was measured in real-time by the Electric Cell-Substrate Impedance Sensing (ECIS) system. Normalized resistance is proportional to the strength of adherence. Resistance measurements were made in every minute and represent the average of three independent wells. Data were analyzed by two-way ANOVA followed by Dunnett’s multiple comparisons (*n* = 3, * *p* < 0.05, ** *p* < 0.01, ns: not significant).

Next, we tested the effect of MP activation on cell adherence accompanying macrophage differentiation. THP-1 cells were differentiated either in the absence or in the presence of H1152 or EGCG for 24, 48, or 72 h, then non-adherent cells were removed, and the total protein content correlating with cell number was measured by sulforhodamine B (SRB) staining (Figure 4A). Absorbance of the PMA-stimulated cells after 72 h was taken as 100%.

Attachment of untreated cells was negligible, while the adherence of PMA-stimulated cells increased over time reaching a maximum after 72 h. The percentage of adherent cells in the presence of H1152 or EGCG was lower at any time point of measurement, and a significant attenuation of adherence was observed at 72 h. To quantify the level of differentiation of THP-1 cells in real-time the Electric Cell-Substrate Impedance Sensing (ECIS) system was applied (Figure 4B). ECIS system measures the changes in resistance between electrodes at the bottom of the culture plate, which is proportional to cell adherence at the surface [29]. Normalized resistance did not change in the case of unstimulated cells, while PMA treatment was accompanied by a gradual increase in resistance, proving the reliability of these measurements as a tool to monitor monocyte to macrophage differentiation. Pretreatment of THP-1 monocytes with H1152 slowed down PMA-induced differentiation and reached much lower level of adherence and unstable macrophage phenotype as indicated by a decrease in resistance after 48 h. In the presence of EGCG, the kinetics of differentiation were initially similar but resulted in weaker strength of adherence, which started to decrease after 48 h. Collectively these data suggest that activation of MP inhibits the efficacy of PMA-induced macrophage differentiation.

## 3. Discussion

Monocyte to macrophage differentiation is a complex process mediated by distinct signaling cascades, which in turn alter downstream transcriptional programs and also have direct influence on certain cellular functions [30]. Protein kinases and phosphatases might also play a vital role in the guidance of macrophage differentiation as important regulators of cellular signaling.

Ser/Thr-specific phosphatases have a well-documented role in the regulation of various cellular processes; however, there are only a few reports demonstrating the involvement of phosphatases in the control of signaling pathways that regulate macrophage differentiation. A series of early studies indicated the relation of the major Ser/Thr-specific phosphatases with VD_3_-induced monocyte differentiation. In these studies VD_3_-stimuli were reported to induce the upregulation of protein phosphatase type 2C (PP2C aka PPM1A) [31] and calcineurin [32], and a subcellular redistribution of PP1 isoenzymes [33] in HL-60 leukemic cells, but the physiological significance and the relevant substrate proteins have remained unidentified. A recent study has verified the role of PP2C as a negative regulator of macrophage differentiation through a mechanism dependent on the downregulation of Akt and STAT1 signaling [34]. Calyculin A, an inhibitor of PP1 and PP2A, augmented the VD_3_-induced differentiation, providing evidence for the implication of these protein phosphatases, too [33]. However, the putative regulatory/targeting subunits for PP1 and PP2A have remained unidentified.

The present study was designed to define the contribution of myosin phosphatase (MP), a PP1-type holoenzyme, to the regulation of monocyte to macrophage differentiation. MP is composed of the δ isoform of PP1 (PP1cδ) and a myosin phosphatase targeting subunit (MYPT1). MYPT1 regulates MP through directing the holoenzyme to its substrates, while phosphorylation of MYPT1 at Thr696 and Thr853 residues results in inhibition of MP activity. MP is known to dephosphorylate MLC20^pS19^ and control actomyosin contractility in smooth muscle and non-muscle cells [35]. Our results have demonstrated that the MP holoenzyme is implicated in the control of monocyte to macrophage differentiation, since both PMA- and VD_3_-induced differentiation of THP-1 cells was accompanied by an increase in the phosphorylation of MYPT1 at the inhibitory phosphosites and a concomitant inhibition of MP. The latter is reflected also in the increased phosphorylation level of MLC20, the major cellular target of MP (see Figure 2 and Figure 3).

Previous studies have demonstrated that PMA-induced macrophage differentiation requires the activation of Rho-associated kinase (ROCK) [12,13]. ROCK is the major effector kinase of small Rho GTPases, and it is involved in the regulation of cytoskeletal processes through the phosphorylation of specific substrates including MLC20, ezrin/radixin/moesin family proteins, and adducin [36]. Dephosphorylation of these proteins is regulated primarily by MP, and a cooperative action of ROCK and MP in the control of cellular processes that involve cytoskeletal rearrangement has been reported by several groups [19,37,38,39]. In contribution to this, our research group has recently demonstrated the implication of ROCK and MP in the mediation of wound healing of epidermal keratinocytes [40]. Our present data show that macrophage differentiation induced by PMA requires not only the activation of ROCK, but a concurrent inhibition of MP too. MLC20-mediated actomyosin rearrangement seems to be essential for monocyte to macrophage differentiation [12]. In agreement with this, we observed a significant increase in MLC20 phosphorylation upon treatment of THP-1 cells with PMA (Figure 3A). This suggests that ROCK activation and MP inhibition are required in order to maintain MLC20 phosphorylation. Inhibition of MP, at least in part, is achieved via phosphorylation of MYPT1 inhibitory sites by ROCK. Accordingly, application of the ROCK inhibitor H1152 prior to PMA stimuli significantly attenuated both MYPT1 and MLC20 phosphorylation, and it impaired the ability of monocytes to differentiate into macrophages, as indicated by lower expression of the macrophage marker CD11b, attenuated proliferation inhibition, and decreased cell adherence.

Many human diseases are affected by an inaccurate monocyte to macrophage differentiation program [2]. For example, excessive macrophage differentiation can augment atherosclerosis, a vascular disease characterized by the formation of atherosclerotic plaques [3]. Thus, pharmacological means to inhibit macrophage differentiation would be valuable to treat atherosclerosis. Indeed, inhibition of ROCK activity by statins or selective ROCK inhibitors has been demonstrated to protect against atherosclerosis [41]; however, the implication of attenuated macrophage differentiation in the anti-atherosclerotic effects of ROCK inhibition has not been investigated. Our findings suggest that drugs that increase the activity of MP to inhibit monocyte to macrophage differentiation could also retard the process of atherosclerosis.

It is a relatively new finding that MP can be activated by potentiating MYPT1 dephosphorylation through the initiation of the 67LR/PKA/PP2A pathway by EGCG [26,27]. Indeed, EGCG has a variety of pharmacological actions including cardiovascular protective effects [42]. Some studies have suggested the effectiveness of EGCG in the treatment of atherosclerosis, but little is known about the underlying molecular mechanism [43]. Wang et al. have demonstrated in PMA-differentiated macrophages that EGCG via 67LR represses the expression of matrix metalloproteinases, which might lead to atherosclerotic plaque stabilization [44]. As revealed in our study, treatment of THP-1 cells with EGCG prior to induction of differentiation effectively reduced MYPT1 phosphorylation, thus activating MP and inhibiting the upregulation of MLC20^pS19^. Considering the important role of MLC20 phosphorylation in macrophage differentiation, our findings might provide new insight into the anti-atherosclerotic effects of EGCG.

In summary, MP inhibition is associated with the control of monocyte to macrophage differentiation. Pharmacological activation of MP either by ROCK inhibitor or EGCG attenuates cell adherence, proliferation inhibition, and differentiation of PMA-stimulated THP-1 cells. Pharmacological activation of MP might be valuable in the treatment of diseases that are coupled with excessive macrophage differentiation.

## 4. Materials and Methods

### 4.1. Reagents and Antibodies

Reagents were obtained from the following sources: H1152, EGCG (Tocris Bioscience, Bristol, UK); protease inhibitor cocktail, sulforhodamine B, PMA, and 1α,25-dihydroxivitamin D_3_ (VD_3_) (Sigma-Aldrich, St. Louis, MO, USA); BCA protein assay (Thermo Fischer Scientific, Waltham, MA, USA). Antibodies and their sources were as follows: MYPT1^pT696^ and MYPT1^pT853^ (Millipore, MilliporeSigma, Burlington, MA, USA); horseradish-peroxidase conjugated anti-rabbit (Sigma-Aldrich, St. Louis, MO, USA); MYPT1 [45]; MLC20^pS19^, p21, PCNA, and horseradish-peroxidase conjugated anti-mouse (Cell Signaling Technology, Danvers, MA, USA); β-actin-HRP, PP1β, and CD11b (Santa Cruz Biotechnology, Dallas, TX, USA).

### 4.2. Cell Culture and Treatments

THP-1 human acute monocytic leukemia cells were purchased from the European Collection of Cell Cultures and maintained according to the supplier’s recommendations. Treatments were carried out at 500,000 cells/mL density in culture medium containing 10% FBS. Cells were pretreated with none or with 10 µM H1152 for 30 min or with 20 µM EGCG for 1 h, then PMA or VD_3_ was added to the media, and the incubation was continued for different time intervals (24–72 h). Cell differentiation was assessed by detecting CD11b surface antigen expression using Western blotting.

### 4.3. Phosphatase Activity Assay

THP-1 cells were added to 24-well plates (5 × 10^5^ cells/well) in culture medium containing 10% FBS and incubated in the presence or absence of 100 nM PMA for 0, 24, or 48 h, then lysates were prepared for phosphatase assay. Cells were washed with phosphate buffered saline (PBS) followed by 0.1 M Tris–HCl (pH 7.6), 150 mM NaCl containing 0.1 mM EDTA (TBS-EDTA), and then collected in 100 μL ice-cold TBS-EDTA supplemented with 0.5% protease inhibitor cocktail, 50 mM 2-mercaptoethanol, 1 mM PMSF, and 3 mM benzamidine. Cells were frozen in liquid nitrogen and then thawed and sonicated, and the lysates were clarified by centrifugation at 16,000× *g* for 10 min. The phosphatase activity of minimally diluted supernatants (~3-fold final dilution in the assays) was assayed with 1 μM ^32^P-labeled 20 kDa light chain (^32^P-MLC20) of turkey gizzard myosin in 20 mM Tris–HCl (pH 7.4) and 0.1% 2-mercaptoethanol at 30 °C for 30 s, and the released ^32^P_i_ was determined as described previously [46]. Phosphatase activities were normalized to the protein content of the samples and expressed as percentages of activities of the untreated samples.

### 4.4. Western Blot Analysis

THP-1 cells were pretreated in 12-well plates (1 × 10^6^ cells/well) with 10 µM H1152 for 30 min or with 20 µM EGCG for 1 h, then with 100 nM PMA or 100 nM VD_3_ for an additional 24 or 48 h. After treatments, cells were lysed in 100 µL RIPA buffer (50 mM Tris-HCl, 150 mM NaCl, 1% Triton X-100, 0.25% Na-deoxycholate, 2 mM EDTA) supplemented with protease inhibitor cocktail and 1 µM microcystin-LR. BCA protein assay was used for measuring protein concentration of the cell lysates. SDS-PAGE and immunoblotting were performed with Mini-Protean system according to Bio-Rad protocol. Membranes were blocked with 5% (*w*/*v*) bovine serum albumin (BSA) solution in Tris-buffered saline (TBS) containing 0.5% Tween 20 (TBST), then incubated overnight at 4 °C with primary antibodies, followed by the application of HRP-conjugated anti-rabbit antibodies. The immunoreactive bands were detected by enhanced chemiluminescence and imaged with the ChemiDoc Touch gel imaging system (Bio-Rad, Hercules, CA, USA). For densitometric analysis of the Western blot images, ImageJ 1.52a software (Wayne Rasband, National Institute of Health, Bethesda MD, USA) was used.

### 4.5. Sulforhodamine B Assay

THP-1 cells were pretreated in 24-well plates (4 × 10^5^ cells/well) with 10 µM H1152 for 30 min or with 20 µM EGCG for 1 h, then with 100 nM PMA for additional 24, 48, or 72 h. Cell culture media along with nonadherent cells were removed at the indicated time points, and adherent cells were washed 3 times with PBS. TCA (50%) was diluted to 10% in serum-free RPMI-1640 and was added to the attached cells for 1 h at 4 °C. Then the supernatant was removed, and the precipitated proteins were washed 5 times with dH_2_O and then air-dried. Cells were stained with 0.4% (*w*/*v*) sulforhodamine B for 10 min, then washed 5 times with 1% acetic acid and air-dried. Bound dye was solubilized with 10 mM Tris-HCl pH 7.4, and absorbance was measured at 515 nm with Multiscan Go microplate spectrophotometer (Thermo Scientific). Absorbance of the PMA-stimulated samples after 72 h was taken as 100%. Histograms represent the mean of three independent experiments.

### 4.6. Electric Cell-Substrate Impedance Sensing (ECIS) Measurement

Electric cell-substrate impedance sensing (ECIS, Applied BioPhysics, Troy, NY, USA) is suitable to measure the impedance of the surface. THP-1 cells were pretreated with none, 10 µM H1152 for 30 min, or with 20 µM EGCG for 1 h, then incubated for an additional 72 h in the absence or presence of 100 nM PMA. Medium of control cells did not contain any added components except the solvent of inhibitors. Resistance measurements were recorded at 4000 Hz per minute in real-time for 72 h. In every experiment set, electric cell-substrate impedance was measured in three independent wells for every treatment.

### 4.7. Statistical Analysis

Results are presented as mean with standard deviation of at least three independent experiments. Statistical analysis of phosphatase activity measurements was performed by multiple t-test and Sidak’s multiple comparisons. Densitometry data of Western blot images was analyzed by two-way ANOVA (followed by Tukey’s or Dunnett’s multiple comparisons, as indicated). Data of sulforhodamine B assay were analyzed by multiple t-test, and ECIS data were analyzed by two-way ANOVA followed by Dunnett’s multiple comparisons using Graph Pad Prism 8.0.1. software. *p* values < 0.05 were considered to be significant.

## Data Availability

Not applicable.
